# Improved Catenated Structures of Bovine Peroxiredoxin III F190L Reveal Details of Ring-Ring Interactions and a Novel Conformational State

**DOI:** 10.1371/journal.pone.0123303

**Published:** 2015-04-23

**Authors:** Zhenbo Cao, Donna P. McGow, Colin Shepherd, J. Gordon Lindsay

**Affiliations:** From the Institute of Molecular, Cell and Systems Biology, CMVLS, University of Glasgow, Glasgow, United Kingdom; University of Edinburgh, UNITED KINGDOM

## Abstract

Mitochondrial 2-cys peroxiredoxin III (PrxIII) is a key player in antioxidant defence reducing locally-generated H2O2 to H2O. A Phe to Leu (F190L) mutation in the C-terminal α-helix of PrxIII, mimicking that found in some bacteria and parasites, increases its resistance to hyperoxidation but has no obvious influence on peroxidase activity. Here we report on the oxidized and reduced crystal structures of bovine PrxIII F190L at 2.4 Å and 2.2 Å, respectively. Both structures exist as two-ring catenanes with their dodecameric rings inclined at 55o to each other, similar to that previously reported for PrxIII C168S. The new higher-resolution structures reveal details of the complex network of H-bonds stabilising the inter-toroid contacts. In addition, Arg123, the key conserved residue, that normally interacts with the catalytic cys (Cp, cys 47) is found in a distinct conformation extending away from the Cp while the characteristic Arg-Glu-Arg network, underpinning the active-site geometry also displays a distinctive arrangement, not observed previously. This novel active-site organisation may provide new insights into the dynamics of the large-scale conformational changes occurring between oxidized and reduced states.

## Introduction

Mitochondria are not only the powerhouses of the cell but also the major intracellular sites of reactive oxygen species (ROS) production [1]. Although ROS are best known for their damaging effects on cellular macromolecules during oxidative stress, there is increasing evidence to indicate that oxidizing agents such as H_2_O_2_ play vital roles in redox signalling [2]. During respiration linked ATP production in the mitochondrial inner membrane, there is significant electron leakage from the electron transport chain, especially from complexes I and complex III, initially generating superoxide anions (O_2_^.-^). However, most superoxide is reduced to H_2_O_2_ by the mitochondrial Mn^2+^-requiring superoxide dismutase (MnSOD). Competitive kinetic studies have also estimated that 90% of mitochondrial H_2_O_2_ is further reduced to water by peroxiredoxin III (PrxIII) within this compartment [3]. Peroxiredoxin V (PrxV), a 1-cys Prx, is also located in mitochondria in addition to other intracellular compartments [4]. Oxidative stress becomes apparent when increased ROS production overwhelms the battery of intra- and extra-mitochondrial anti-oxidant defence systems.

**Fig 1:**
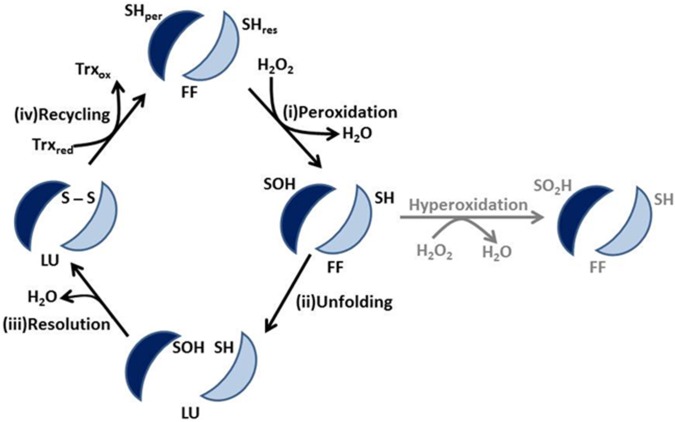
Schematic illustration of various states of PrxIII during the reaction cycle. The homodimer of the PrxIII dodecamer represents a functional unit during the reaction cycle: (i) the peroxidatic cysteine (SH_per_) reduces hydrogen peroxide and is converted to its sulfenylated (SOH) form. (ii) the Cp loop housing the peroxidatic cysteine unfolds from its FF to LU conformation. (iii) the peroxidatic cysteine forms a disulfide bond with the resolving cysteine eliminating an H_2_O molecule (iv) mitochondrial thioredoxin (Trx_red_) reduces the disulfide bond to regenerate the reduced active-site cysteine while the Cp loop re-assumes the FF state conformation. The sulfenylated cysteine intermediate can be further oxidized to its sulfinylated or sulphonylated forms while it remains in the FF state at elevated H_2_O_2_ levels.

PrxIII is a prominent member of the ubiquitous peroxiredoxin family that function as thiol-dependent peroxidases with dual roles in anti-oxidant protection and redox signalling in eukaryotes [5,6]. In mammalian cells, PrxI and II reside in the cytoplasm, PrxIII is mitochondrially-located and PrxIV is confined to the endoplasmic reticulum. Like other typical 2-Cys Prxs, PrxIII employs its peroxidatic, active-site cysteine (Cys47) to react with hydrogen peroxide forming cysteine sulfenic acid (CysOH) [7] (Fig 1). The resolving cysteine (Cys168) from the adjacent monomer then forms a disulfide bond with the peroxidatic cysteine releasing an H_2_O molecule. The resulting disulfide is reduced by mitochondrial thioredoxin (Trx2) that is itself reduced by a mitochondrial NADP-linked thioredoxin reductase (TrxR2). The sulfenic cysteine of mammalian Prxs can be inactivated during times of oxidative stress by further oxidation to sulfinic acid (Cys-SO_2_H) and even sulfonic acid (Cys-SO_3_H). These inactive, hyperoxidised Prx species are considered to be integral players in H_2_O_2_-mediated signalling [6,8]. Inactive cytosolic mammalian Prxs, in the cysteine sulfinic acid state, can be re-reduced by sulfiredoxin (Srx) and ATP [9] whereas the CysSO_3_H Prx state is thought to be damaged irreversibly [10]. It has also been reported that Srx can be translocated from the cytosol to mitochondria in response to oxidative stress [11].

Structural analysis has shown that Prxs undergo a large conformational change during the transition from oxidized to reduced states [12]. Depending on whether the peroxidatic cysteine (Cp) is reduced or disulfide-bonded, the active site is either in the fully folded (FF) or locally unfolded (LU) conformation. The two catalytic cysteines are separated by 13 Å in the FF state while they require to be in close proximity to enable disulfide bond formation on oxidation. Thus, during the catalytic cycle, the Prx structure will alternate rapidly between FF and LU states requiring large-scale movement of the Cp loop.

It has been established that the YF motif in the C-terminal α-helix is involved in promoting the hyperoxidation step by delaying the conformational change from FF to LU [6]. The YF motif is absent in most prokaryotic Prxs that are generally resistant to hyperoxidation and function exclusively in combating oxidative damage. However, a similar YL motif is present in some bacteria [13] and parasites [14] although its involvement in regulating the sensitivity of these Prxs to H_2_O_2_-induced inactivation has not been studied to date.

Prxs have a remarkably high catalytic efficiency towards H_2_O_2_ with second order rate constants of ~ 10^7^ M^-1^ s^-1^ [15]. This is linked to the microenvironment around the active site lowering the pK_a_ of the cysteine to provide an optimised substrate binding site [16]. The Cp-thiolate is stabilised and activated for nucleophilic attack on its peroxide substrate by hydrogen bonding to a conserved Arg residue (Arg123) and a backbone amide group [16,17]. These authors also concluded that an Arg–Glu–Arg hydrogen-bonding network leads the guanidinium group of the active-site Arg to be in position to support the Cp. Kinetic and computational studies have revealed that, not only Arg123 but also Arg146 in human PrxIII, is equally vital for activity and it has been proposed that both Arg residues interact directly with substrate [17].

Although there is an underlying basic functional dimeric unit, typical 2-Cys Prxs exist as higher order toroidal structures. In most cases, they form decameric rings although octamers and dodecamers have also been observed [18,19]. The transition from dimers to toroids is known to be redox and concentration dependent in most cases [20]. The ring structure can further assemble into more complex quaternary states. Jang *et al* have reported an association of the PrxII decamer into a dodecahedral structure on hyperoxidation *in vitro* [21]. Moreover, a TEM study of bovine PrxIII has demonstrated a tendency for individual dodecameric toroids to stack end to end, forming long filaments (40–50 rings) in the case of the C47S mutant [7]. Remarkably, the 3.3 Å crystal structure of bovine PrxIII C168S has revealed a quaternary organisation consisting of interlocking dodecameric rings forming a two-ring catenane [22]. Transmission electron microscopy (TEM) analyses of PrxIII samples in dilute solution before and after crystallization have demonstrated that the population consists largely of single toroids together with 3–5% double interlocked rings suggesting that catenane formation is a dynamic process and not simply an artefact of crystal packing [20].

In this paper, we report on the crystal structures of both oxidized and reduced forms of bovine PrxIII F190L to 2.4 Å and 2.2 Å, respectively and examine some of its novel structural and enzymatic properties.

## Results

### Activity of PrxIII F190L and its resistance to hyperoxidation

**Fig 2:**
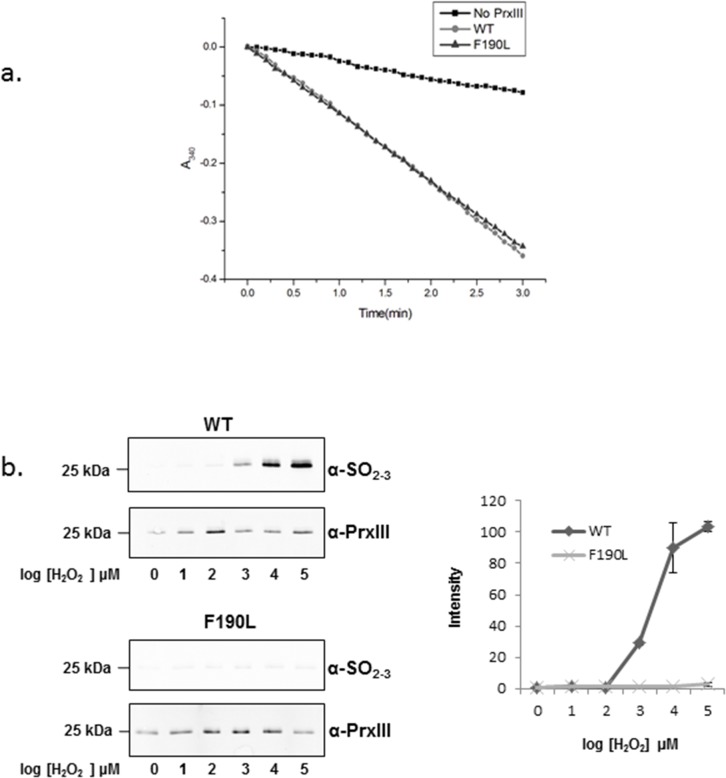
The effect of F190L to peroxidase activity and sensitivity to hyperoxidation. **a.** Time course of PrxIII dependent NADPH oxidation coupled to H_2_O_2_ reduction for PrxIII wild-type and F190L. NADPH oxidation was monitored at 340 nm in a 1 ml reaction mixture containing with 5 μM of PrxIII, 2.1 μM Trx, 1.5 μM TrxR2 and 0.5 mM NADPH in 50 mM NaH_2_PO4 buffer, pH 7.4 containing 150 mM NaCl, 1 mM EDTA. A reaction mix without PrxIII was used as the negative control. This is a representative result from three independent experiments with similar results. **b.** Dose-dependent increase in PrxIII wild-type and F190L mutant hyperoxidation after exposure to hydrogen peroxide at the indicated concentrations. PrxIII hyper-oxidation was monitored by western blotting using an antibody that recognizes primarily the sulfinic or sulfonic acid forms of Prxs (α-SO_2-3_). The assay was carried out in the presence of 1 mM DTT as reductant. Western blots were used for quantification of hyperoxidized PrxIII by densitometry. Intensity of hyper-oxidized PrxIII was normalized against loading control blotted with an anti-His antibody. Error bars represent the mean ±S.D. from three independent experiments.

Mammalian PrxIII contains a highly-conserved YF motif located in its C-terminal α-helix that is responsible for its susceptibility to hyperoxidation. In contrast this region is poorly conserved in prokaryotes although some bacteria and parasites have a similar YL motif of unknown function. To evaluate the possible role of the poorly-characterised YL motif, an F190L PrxIII mutant was generated from native bovine PrxIII. Firstly the intrinsic peroxidase activity of PrxIII F190L was assessed and found to be identical to the wild-type enzyme (Fig 2A). Wild-type and mutant PrxIII hyperoxidation status was also determined with an antibody that primarily recognizes Prx active-site CysSO_2_ and CysSO_3_ moieties [23]. During exposure to increasing H_2_O_2_, both PrxIIIs were recycled in the presence 1mM dithiothreitol (DTT). The mutant was found to be relatively insensitive to hyperoxidation even at high peroxide levels as compared to the wild-type control (Fig 2B). These data highlight the importance of the large Phe 190 residue in conferring overall PrxIII sensitivity to hyperoxidation while having no obvious effect on enzymatic function.

### Reduced bovine PrxIII F190L is present as a two-ring catenane in the crystal structure

**Fig 3:**
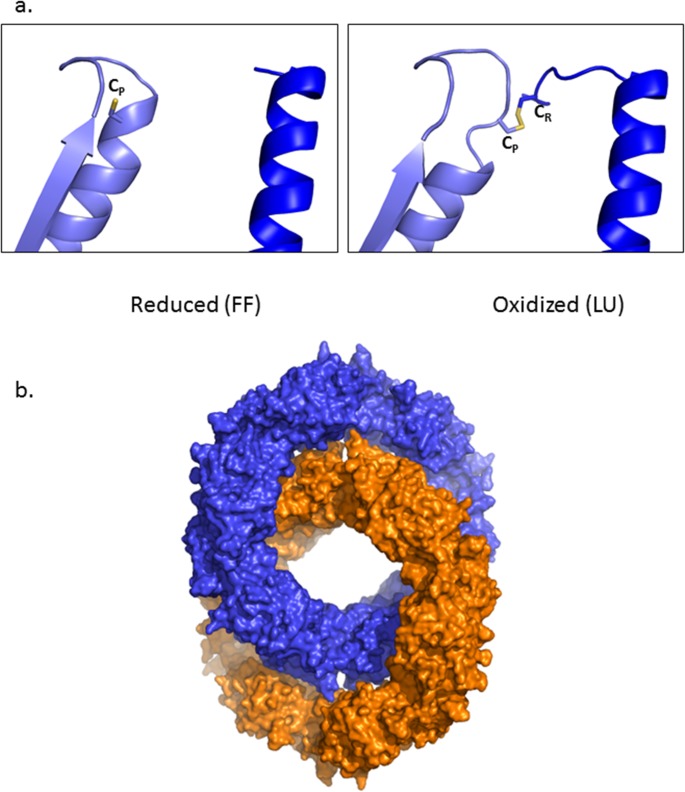
PrxIII forms interlocked rings in both oxidized and states. a. Detailed cartoon diagram of reduced and oxidized PrxIII F190L showing the transition between fully folded (FF) and locally unfolded (LU) forms. The active-site cysteine sulfur atom is shown as yellow sticks. Different chains are colored in cyan and blue, respectively. b. Surface diagram of the reduced PrxIII F190L catenane showing two interlocking dodecameric rings in orange and blue, respectively. Both oxidized and reduced states of the protein display a very similar catenaned structure.

In the presence of 1mM DTT, reduced PrxIII F190L crystallized with a P2_1_2_1_2 space group. The data-processing and refinement statistics for the PrxIII F190L model are listed in Table 1. Twelve monomers are present in the asymmetric unit forming a toroidal structure. As reported for PrxIII C168S, the peroxidatic cysteine (Cys47) is located at the end of a long helical segment similar to that adopted in the FF conformation in typical 2-cys Prxs although the C-terminal region including the resolving cysteine (Cys168) appears to be unstructured in these cases (Fig 3A).

**Table 1:** Data collection and refinement statistics.

	Oxidised	Reduced
PDB code	4MH3	4MH2
Data collection		
Space group	P2_1_2_1_2	P2_1_2_1_2
Unit cell (Å)	a = 142.89 b = 290.88	a = 139.57 b = 260.82
	c = 81.14	c = 81.66
Resolution (Å)*	102.06–2.4(2.53–2.4)	93.35–2.2(2.32–2.2)
No. of unique observations*	130007(17808)	149669(21040)
Multiplicity*	4.1(3.5)	6.1(5.1)
Completeness(%)*	98.2(93.9)	98.7(96.3)
Mean I/σ*	11.3(1.8)	11.4(1.6)
R_merge_(%)*	9.5(73.2)	8.9(73.8)
Wilson plot B-factor (Å^2^)	55.8	38.2
Refinement		
R_work_/R_free_ (%)	18.2/22.1	18.1/21.9
rmsd for bond lengths (Å)	0.012	0.012
rmsd for bond angles (deg)	1.58	1.50
Ramachandran plot (%)		
Favored	95.61	97.02
Allowed	100.00	100.00

*Values in parentheses are for the highest-resolution shell.

Overall, there are 6 homodimers interacting by hydrophobic contacts to complete an (α_2_)_6_ dodecameric toroid. Interestingly, PrxIII F190L is also present as 2 physically interlocked dodecameric rings—a two-ring catenane (Fig 3B), as described previously for the C168S mutant [22]. Thus, in several respects, PrxIII does not behave as a typical 2-cys Prx. These include a distinct C-terminal region that renders PxIII less sensitive to hyperoxidation than other typical 2-cys Prxs [24], and a unique ability to form dodecameric toroids (rather than decamers) with a large central cavity that favors further assembly into two-ring catenanes.

### Ring-ring interactions

As indicated, the most remarkable feature of the current structure is its presence as a two-ring catenane consisting of two interlinked dodecameric rings. Each (α_2_)_6_ toroid has an external diameter of 150 Å enclosing a large 70 Å central cavity. The two rings are inclined at an angle of 55° allowing large areas of contact between the dodecamers.

**Fig 4:**
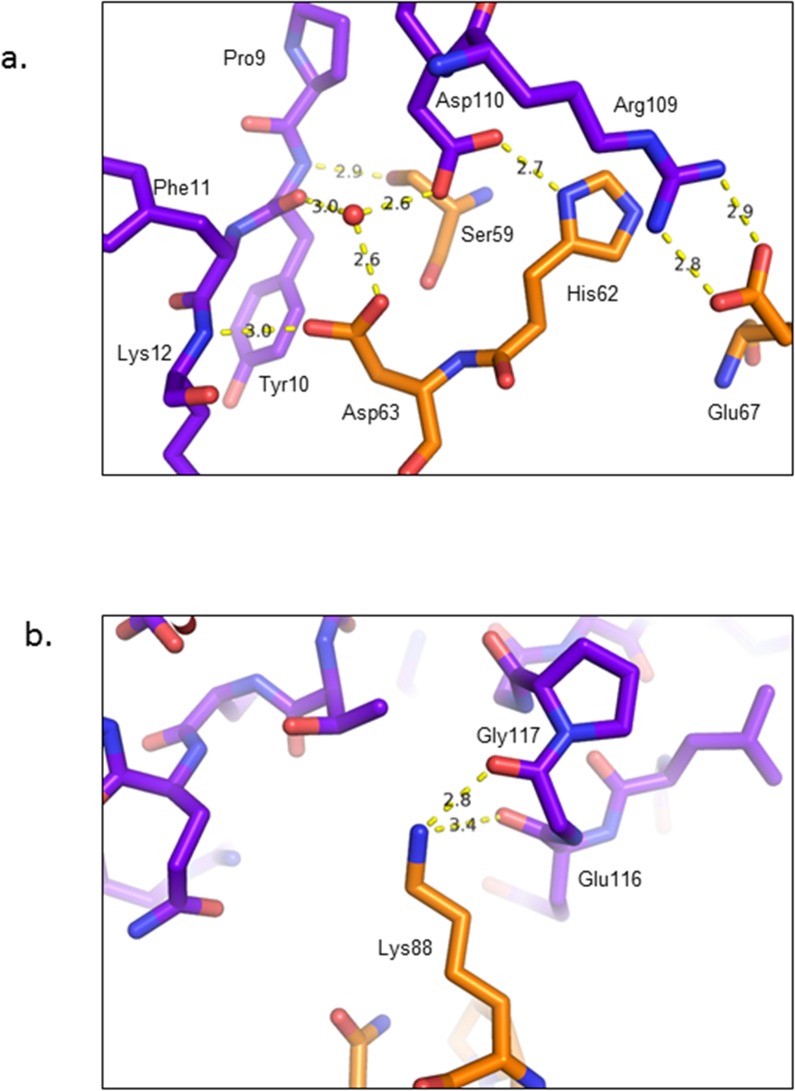
Ring-ring interactions. Detailed representation of the hydrogen bonding network in different contact areas between the rings. The two rings are colored in blue and orange, respectively. The hydrogen bonds are shown in dotted yellow lines with the distance in angstroms (Å).

In this higher resolution 2.2 Å structure, a detailed picture is revealed for the first time of the precise nature of the ring-ring interactions stabilising the catenated state. The major inter-toroidal contact area comprises a network of hydrogen bonds between residues Ser59, His62, Asp63 and Glu67 from chain A of ring 1 and Pro9, Tyr10, Phe11, Lys12, Arg109 and Asp110 from chain H of ring 2 (Fig 4A). Specifically, the backbone N of Tyr10 interacts with the side chain hydroxyl of Ser59. Similarly, the Lys12 amide N binds to the carboxyl group of Asp63 while a water molecule links the carboxyl groups of Asp63 and 110 with the amide N of Phe11. In addition, the Asp110 carboxylate also forms a contact with the imidazole N of His62. Finally the guanidinium group of Arg109 forms 2 hydrogen bonds with the carboxyl group of Glu67. A second region of inter-ring hydrogen bonds is observed between the ε-amino group of Lys88 from chain A of ring 1 and the carboxyl groups of Glu116 and 117 from chain G of ring 2 (Fig 4B). In total, there are 8 areas of inter-toroid contact arising from the presence of four non-crystallographic symmetry related copies of 2 distinct interfaces. A potential third site of interaction involving hydrogen bond formation between Lys12 from chain C and Tyr10 from chain J, as observed for the C168S mutant, is absent in the current structure.

### Locally unfolded conformation of oxidized PrxIII

In the absence of DTT, PrxIII was crystallized in the same space group as for the reduced form but in a slightly different unit cell. This 2.4 Å structure shows all the major structural and organisational features of its reduced counterpart. In this case, the Cp loop around Cys47 adopts the locally unfolded (LU) conformation (Fig 3B) and the disulfide bond between the Cys47 of one monomer and Cys168 from its companion subunit is visible while the residues beyond Pro169 are not traceable. Although in dilute solution, the oxidized PrxIII toroid is much less stable than the reduced species showing significant dissociation into dimers, it still exists as dodecamers in the crystal structure, indicating that oligomer formation is concentration-dependent as reported previously (19). Most strikingly, oxidized PrxIII also forms a two-ring catenane that is almost identical to the reduced form with the planes of the rings again inclined at 55^0^ to each other.

In addition, the two major regions of inter-chain contact in oxidised PrxIII F190L described above are identical to those in the reduced structure. However, the third potential point of interaction between Lys12 and Tyr10, observed previously in the C168S mutant and absent in reduced PrxIII F190L, is again evident in its oxidized counterpart where these two residues are in close proximity, enabling hydrogen bond formation.

### Arg123 exhibits a distinctive, previously-unobserved conformation

As described before, Arg123 of PrxIII (or its equivalent) is highly-conserved in typical 2-Cys peroxiredoxins and plays an important role in supporting peroxidase activity. Point mutations in Arg123 or Arg146 located 3–4 Å and 6–7 Å from the active site Cys47 respectively, each lead to a decrease in catalytic power by 5 orders of magnitude. Double mutants show a further 100-fold loss of peroxidase function suggesting a cooperative role for these two Arg residues [17].

**Fig 5:**
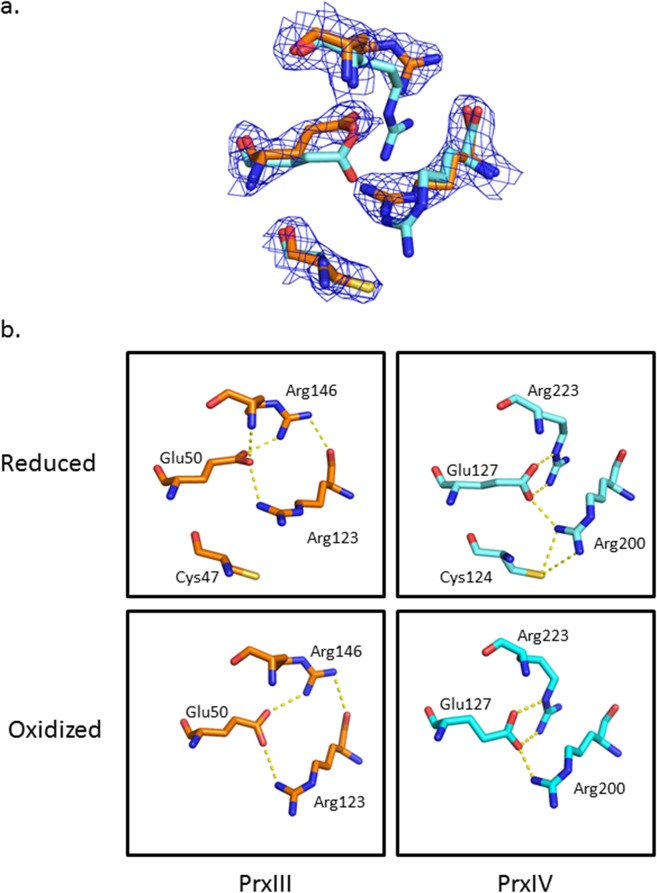
Arg123 and the Arg-Glu-Arg network are in a new conformational state. **a.** Structure alignment between reduced PrxIII F190L (orange) and PrxIV C51A (cyan) showing Arg123, Glu50 and Arg146 are in different positions. The position of the peroxidatic cysteine is fixed. The 2Fo-Fc electron density map around the residues of PrxIII is contoured at 1.0 σ. **b.** Detailed representation of the hydrogen bonding network in both reduced and oxidized PrxIII and PrxIV from the same viewpoint. Hydrogen bonds are shown in dotted yellow lines.

In previous publications, e.g. on the reduced (FF) structure of PrxIV [23], the guanidinium group of the major Arg residue (Arg200) has been shown to interact directly with the peroxidatic cysteine sulfur lowering its pK_a_, thereby favoring thiolate formation and greatly enhancing its reactivity. This key Arg residue maintains an identical conformation after conversion of PrxIV to the oxidized (LU) state that is accompanied by large-scale movement of the peroxidatic and resolving cysteines. In contrast, with PrxIII F190L in its reduced state, the conformation of this conserved Arg is quite distinct; instead of facing the active-site thiolate, the Arg side chain adopts an average conformation inclined away from the Cp sulfur to a distance of 4–5 Å (Fig 5A). Interestingly, however, individual active-site geometries around the 2-ring catenane display considerable microheterogeneity in this region with the Arg side chain adopting a variety of positions ranging from 3.4 Å to 5.2 Å from the cysteine sulfur. These data strongly suggest that the Arg side chain is highly mobile and can move away readily from its interaction with the cysteine thiolate during the initial phase of the catalytic cycle.

### Distinctive features of the Arg-Glu-Arg network in PrxIII

A further interesting difference relates to the organisation of the Arg-Glu-Arg network, first reported by Hall *et al* [16], in which the conserved second Arg in the typical 2-Cys Prxs (Arg223 in PrxIV) contributes to the positioning of the active-site first Arg (Arg200 in PrxIV) via a series of hydrogen bonds, establishing a basis for their cooperative interactions as suggested from mutation analysis (Fig 5B). A similar but distinctive hydrogen-bonding network is also present in the PrxIII structure; however, the locations of all three key residues are different from other Prxs as is the nature of the bonding network itself. In our PrxIII structure (reduced), the main chain carbonyl of Arg123 interacts directly with the guanidino group of Arg146 in addition to both residues interacting indirectly via Glu50. In contrast, no direct interaction between these 2 key Arg moieties is apparent in the equivalent PrxIV structures. In addition, Arg 146 is too distant to have direct contact with bound substrate or the active-site cysteine as proposed by Nagy *et al* [17]. Inspection of the Arg-Glu-Arg network in the oxidised PrxIII structure again reveals the presence of a hydrogen bond between the main chain carbonyl of Arg123 and guanidino side chain of Arg146 (Fig 5B). However, the hydrogen bonds linking Arg123 to Glu50 are altered as a result of the extension of the Arg123 side chain towards the location occupied by the active-site Cys47 in the reduced state. In direct contrast, the reported geometry and hydrogen bonding arrangements of the Arg-Glu-Arg network in PrxIV are identical in both oxidized and reduced forms [23].

## Discussion

The YF motif in the C-terminal helix of mammalian 2-cys Prxs, including PrxIII, has been shown to be integral to establishing its susceptibility to hyperoxidation. It has been proposed that the role of this helix is to cover the active site and to limit the dynamic movement of the Cp loop [6]. In the F190L mutant, this helix is established to be destabilised allowing greater access to the active-site thiolate and more rapid disulfide bond formation. As a consequence, its capacity to react with a second molecule of H_2_O_2_ is greatly diminished, highlighting the importance of this conserved Phe residue in regulating the hyperoxidation status of the peroxidatic cysteine. The mutant enzyme, however, displays identical peroxidase activity to the wild-type, indicating that limitation in the rate of movement of the peroxidatic loop, while leading to increased susceptibility to hyperoxidation, is not the rate-limiting step in the peroxidation reaction, at least under *in vitro* assay conditions.

Surprisingly, in our case, it has proved impossible to locate the positions of the C-terminal residues beyond residue 164 that are thought to be rather stable in other peroxiredoxins in the reduced state. However, the C-terminal region of human PrxIII has also been shown to influence its susceptibility to hyperoxidation [24]. Selective substitution of several C-terminal residues in PrxIII making it more similar to a PrxII-like protein further increased its sensitivity to hyperoxidation. This observation together with the lack of electron density of the C-terminal region in the reduced forms of bovine PrxIII C168S may suggest that the PrxIII C-terminus is not as ordered as in other 2-Cys Prxs. Interestingly, the Cp loop of chains F and G of the reduced form had to be modelled in the dual conformation with half in FF and half in LU. The existence of both LU and FF states at these specific sites may be the result of DTT oxidation or X-ray radiation directly promoting generation of ROS leading to partial oxidation. For chains F and G, Arg123 in the FF state was located in relatively close proximity (3.4–3.5Å) to Cys47 as expected if it was returning initially to a position in the vicinity of the active-site cys immediately following its re-reduction (Fig 4). Interestingly, a dual conformation of the Cp loop has also been modelled in the human Prx IV structure [25].

In previous studies, the conserved Arg has been shown to be present in only two highly similar conformations, both with the Arg pointing towards the Cp [16]. Here, our structure reveals a new and distinctive Arg123 conformation with its side chain inclined away from the active-site Cys. In this orientation, Arg123 still forms good hydrogen bond contact with the adjacent Glu50 and Arg146 residues and is strongly supported by the electron density map (Fig 5A). The altered organisation of the Arg-Glu-Arg hydrogen-bonding network in the oxidized and reduced states also demonstrates that these interactions can be more flexible than previously reported. In addition, the new Arg123 conformation, together with the lack of C-terminal structure and the dual conformation of the Cp loop at two specific sites suggests that our structure is not in the typical fully-reduced form, but may well represent a trapped intermediate state. The most striking feature of the crystal structure of the PrxIII C168S mutant is its existence as 2-interlocking dodecameric rings. The PrxIII F190L mutant also forms a similar structure, irrespective of its redox state. This remarkable quaternary organisation appears to be a unique feature of bovine PrxIII since no other similar Prx structure has been reported to date.

Sequence alignment of PrxIII with PrxI, II and IV reveals that the key residues involved in the ring-ring interactions are not well conserved (S1 Fig). Thus while the residues equivalent to Arg109 and Asp110 are conserved in most cases as are the amino acids corresponding to residues 9–12 in PrxIII, non-conserved substitutions at positions equivalent to Ser59, His62 and Asp63 in PrxIII disrupt the potential for any possible interaction between monomers. For example, Ser59, His62 and Asp63 in PrxIII are replaced by their counterparts Glu136, Arg139 and Ser140 in PrxIV. A further notable feature is that the inner diameter of the central cavity is larger in PrxIII than Prxs I, II and IV (70Å versus 60Å respectively). The increased dimensions of this cavity in PrxIII together with the unique hydrogen bonding arrangements that can occur between adjacent monomers located at the interfaces of interlinked toroidal dodecamers appear to be responsible for its exclusive ability to form 2-ring catenanes.

As reported previously [20], formation of these 2-ring catenanes is a dynamic concentration-dependent and reversible process as only a small proportion of double toroids (3–5%) are visible by TEM in dilute solution before crystallisation or after crystal dissolution with the reminder present as single toroids. Although its physiological relevance, if any, has still to be established, it is probable that the catenated form is the predominant state in the crowded environment of the mitochondrial matrix where protein concentrations are reported to be approx. 200mg/ml.

Interestingly, the recent discovery of a new enzyme for CS_2_ conversion to H_2_S and CO_2_ in the acidothermophile, *Acidianus A1-3* has revealed the underlying molecular basis for its existence as an unusual catenated, hexadecameric oligomer, that is responsible for some of its novel enzymatic properties [26].

The ability of Prxs to form large supramolecular assemblies, including nanotubes, has been exploited recently employing a Prx1 mutant as a molecular template to promote metal-induced self-assembly of one dimensional nanoscopic structures housing linear arrays of Ni^2+^-functionalised gold nanoparticles in their central cavities [27]. Formation of ordered arrays of protein-metal complexes is increasingly being exploited in the assembly of electronic nanodevices for a variety of technological applications. The unique ability of PrxIII to form 2-ring catenanes may similarly prove advantageous in the future development of advanced functional materials.

## Materials and Methods

### Mutagenesis, Protein Purification and Crystallization

Specific mutagenic oligonucleotide primers were designed to the region of the gene containing Phe190 to permit its conversion to leucine. Site-directed mutagenesis of bovine PrxIII was performed using the QuikChange™ Site-directed Mutagenesis Kit (Stratagene) according to the manufacturer's instructions. As bovine PrxIII, lacking its N-terminal 62 amino acid mitochondrial targeting signal, was employed for these studies, residue 1 refers to the N-terminal alanine of the mature protein. The N-terminally His-tagged bovine PrxIII F190L construct housed in pET14b was overexpressed for 5h at 22°C in *E*. *coli* BL21 pLysS cells prior to disruption by French pressure treatment. Purification was performed by zinc-resin affinity chromatography followed by gel filtration chromatography (HiPrep 16/60 Sephacryl S-300, GE healthcare) in 50 mM NaCl, 20 mM HEPES (pH 7.2), 5 mM EDTA, with and without 10 mM dithiothreitol (DTT). Prior to crystallization, the protein was concentrated to a final concentration of 15 mg ml^-1^.

Crystallization trials were performed at 16°C by using the sitting-drop vapor-diffusion method. Brick-like crystals of the reduced form obtained from drops composed of 2 μl protein solution mixed with a 2 μl volume of a 1 ml reservoir solution containing 34% (v/v) 2-methyl-1,3-propanediol, 0.1 M phosphate-citrate pH 4.2, grew to a size of 0.5 × 0.2 × 0.2 mm in a week. Rod-like crystals of the oxidized form obtained from drops composed of 2 μl protein solution mixed with a 2 μl volume of a 1 ml reservoir solution containing 36% (v/v) 2-methyl-1,3-propanediol, 0.1M phosphate-citrate pH 4.1, grew to a size of 1.0 x 0.1 x 0.1 mm in a week.

### Data Collection

The mother liquor with 25% (v/v) glycerol was used as a cryoprotectant. X-ray diffraction data sets were collected at 100K by using synchrotron radiation (Diamond, UK) at the I02 beamline equipped with an ADSC Q315r CCD detector. A total of 720 frames were recorded using 0.5° of oscillation with a crystal detector distance of 340 mm and 407.4 mm, for reduced and oxidized forms respectively. Data were processed to a resolution of 2.2 Å for the reduced form and 2.4 Å for the oxidized form by using the programs MOSFLM, SCALA, and TRUNCATE from the CCP4 package [28]. Data collection statistics are summarized in Table 1.

### Structure Determination

An initial phase set was obtained by molecular replacement with the program PHASER [29] by using a dimer of PrxIII C168S (PDB code 1zye) as a search model to locate six dimers. This gave a solution of a circular, hexameric assembly of dimers. By applying crystallographic two fold symmetry, two interlocked dodecameric ring structures were found in one unit cell. This structure was refined by ten cycles of rigid-body refinement, with a dodecameric ring treated as 12 domains, by using the program REFMAC in the CCP4 suite [28]. Structural modelling was performed with *Coot* [30]. The TLS thermal mode was used, thereby allowing a separation of the overall lattice vibrations before the standard restrained refinement of atomic coordinates and the individual atom isotropic *B*-factors [28]. Molprobity was used to monitor the model geometry[31]. All figures were drawn by PyMOL (Version 1.4, Schrödinger, Camberley, UK).

### Hyperoxidation assay

Wild type or PrxIII F190L (5μM) was mixed with 1mM DTT as reductant, various concentrations of H_2_O_2_ were added and samples incubated for 10 min at room temperature. An equal amount of sample buffer containing 100 mM dithiothrietol (DTT) was added before boiling for 5 min. The extent of hyperoxidation of PrxIII was assayed by western blot analysis using an antibody that recognizes the sulfinylated or sulfonylated forms of Prxs (α-SO_2-3_). After SDS-PAGE separation, proteins were transferred to nitrocellulose membrane (GE Healthcare). Nonspecific binding was blocked using 5% (w/v) nonfat dried skimmed milk in TBST buffer (50mM Tris buffer, pH 7.5, containing 150mM NaCl and 0.1% (v/v) Tween 20). Primary antibody (α-SO_2-3_ for hyperoxidised form of Prxs and α-His for loading control) was diluted 1:1000, and incubations were performed for 16 h at 4°C. Secondary antibody was diluted 1:5000 in TBST buffer, and incubations were performed in a lightshielded box for 45 min at room temperature. Specific proteins were visualized using an Odyssey SA imaging system (LI-COR Biosciences). For quantification of fluorescent Western blots, scans were performed at minimum intensities required to detect all relevant proteins. Densitometry was then performed on unmodified output images using ImageJ (National Institutes of Health).

### Peroxidase activity assay

Peroxidase activity was measured by monitoring NADPH oxidation as described previously [20]. In short, a 1 ml assay mix containing 2.1 μM Trx2, 1.5 μM TrxR2 and 0.5 mM NADPH in 50 mM NaH_2_PO4 buffer, pH 7.4, 150 mM NaCl and 1 mM EDTA was incubated at 25°C. The reaction was initiated by adding 0.1 mM H_2_O_2_. The A_340_ was recorded on an Ultrospec 4300 pro UV/Visible spectrophotometer over 180s to monitor the oxidation of NADPH as the reducing substrate donor.

### Accession numbers

Coordinates and structure factors have been deposited in the Protein Data Bank with accession number: **4MH2, 4MH3**

## Supporting Information

S1 Fig Sequence alignment of typical 2-cys Prxs. Sequence alignment of human PrxI to PrxIV and bovine PrxIII showing difference between Prxs. Black arrows indicate the residues involved in bovine PrxIII ring ring interactions.
(PPTX)
