# Supplementary material for: Improved Catenated Structures of Bovine Peroxiredoxin III F190L Reveal Details of Ring-Ring Interactions and a Novel Conformational State
Source: PLoS One. 2015 Apr 23;10(4):e0123303. doi: 10.1371/journal.pone.0123303 (PMC4407889; doi:10.1371/journal.pone.0123303)
Supplement: S1 Fig — Sequence alignment of human PrxI to PrxIV and bovine PrxIII showing difference between Prxs. Black arrows indicate the residues involved in bovine PrxIII ring ring interactions. (PPTX) [file pone.0123303.s001.pptx]

## Slide 1
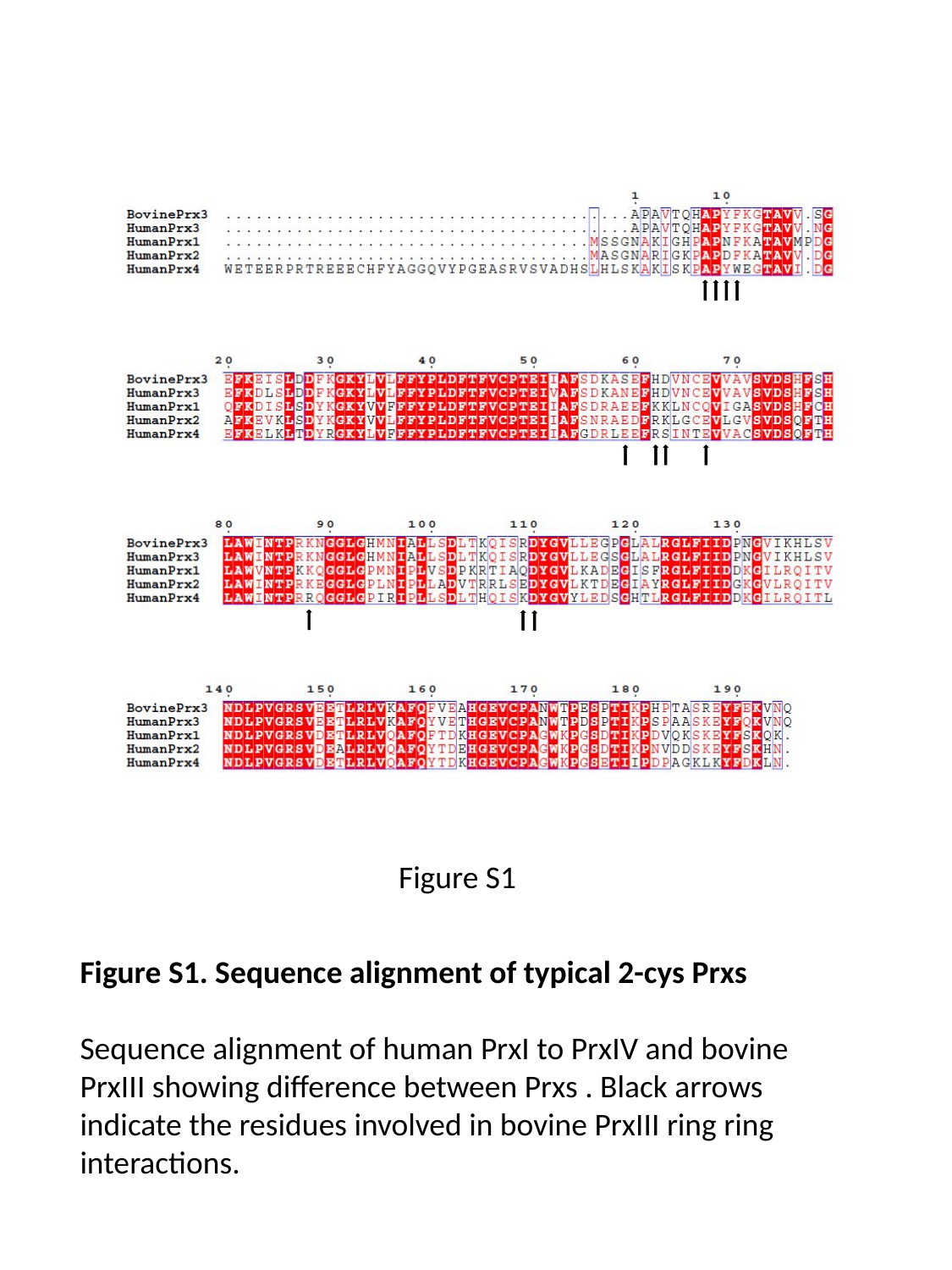

Figure S1
Figure S1. Sequence alignment of typical 2-cys Prxs
Sequence alignment of human PrxI to PrxIV and bovine PrxIII showing difference between Prxs . Black arrows indicate the residues involved in bovine PrxIII ring ring interactions.
